# Volatilomic insight of head and neck cancer via the effects observed on saliva metabolites

**DOI:** 10.1038/s41598-018-35854-x

**Published:** 2018-12-07

**Authors:** Ravindra Taware, Khushman Taunk, Jorge A. M. Pereira, Amey Shirolkar, Dharmesh Soneji, José S. Câmara, H. A. Nagarajaram, Srikanth Rapole

**Affiliations:** 1 Proteomics Lab, National Centre for Cell Science (NCCS), Ganeshkhind, SPPU Campus, Pune, 411007 India; 2 CQM – Centro de Química da Madeira, Universidade da Madeira, Campus Universitário da Penteada, Funchal, 9000-390 Portugal; 3 Malignant Disease Treatment Centre, Military Hospital (Cardio Thoracic Centre), Armed Forces Medical College, Pune, 411040 India; 4 Faculdade de Ciências Exatas e da Engenharia, Universidade da Madeira, Campus Universitário da Penteada, Funchal, 9000-390 Portugal; 5 Laboratory of Computational Biology, Centre for DNA fingerprinting & Diagnostics (CDFD), Nampally, Hyderabad, 500001 India; 6 Department of Biotechnology & Bioinformatics, School of Life Sciences, University of Hyderabad, Hyderabad, 500046 India

## Abstract

Head and neck cancer (HNC) is a heterogeneous malignant disease with distinct global distribution. Metabolic adaptations of HNC are significantly gaining clinical interests nowadays. Here, we investigated effects of HNC on differential expression of volatile metabolites in human saliva. We applied headspace solid phase microextraction coupled with gas chromatography-mass spectrometry analysis of saliva samples collected from 59 human subjects (HNC − 32, Control − 27). We identified and quantified 48 volatile organic metabolites (VOMs) and observed profound effects of HNC on these metabolites. These effects were VOM specific and significantly differed in the biologically comparable healthy controls. HNC induced changes in salivary VOM composition were well attributed to *in vivo* metabolic effects. A panel of 15 VOMs with variable importance in projection (VIP) score >1, false discovery rate (FDR) corrected *p*-value < 0.05 and log_2_ fold change (log_2_ FC) value of ≥0.58/≤−0.58 were regarded as discriminatory metabolites of pathophysiological importance. Afterwards, receiver operator characteristic curve (ROC) projected certain VOMs viz., 1,4-dichlorobenzene, 1,2-decanediol, 2,5-bis1,1-dimethylethylphenol and E-3-decen-2-ol with profound metabolic effects of HNC and highest class segregation potential. Moreover, metabolic pathways analysis portrayed several dysregulated pathways in HNC, which enhanced our basic understanding on salivary VOM changes. Our observations could redefine several known/already investigated systemic phenomenons (e.g. biochemical pathways). These findings will inspire further research in this direction and may open unconventional avenues for non-invasive monitoring of HNC and its therapy in the future.

## Introduction

Head and neck cancer (HNC) is a heterogeneous malignant condition characterised by tumours arising from the mucosal lining of oral cavity, facial sinus, salivary glands and pharynx^1^. HNC is one of the most common cancers across the globe and collectively contributed to 529,500 incidences and caused 292,300 fatalities in 2012^2^. Especially in Indian subcontinent, tobacco and alcohol habits along with infections of human-pappilomavirus (HPV) and epstein-bar virus (EBV) are the major aetiology for high prevalence and malignancy of HNC^3–5^. Although, open biopsy is the only confirmatory diagnostic test used within a high-risk population, monitoring of HNC is rather compromised due to low availability of reliable clinical biomarkers^6^ and leads to the poor survival outcomes of HNC cases^7–9^. Due to its critical pathophysiology and ubiquitous manifestations, systemic/metabolic effects of HNC gained significant clinical importance during recent years.

Analysis of volatile metabolites is a steadily rising field of multidisciplinary metabolomics in relation to physiological^10–13^, pathophysiological^14–17^ and therapeutic^18–20^ monitoring. In this perspective, non-invasive assessment of metabolic effects e.g. via salivary metabolites may enhance our conventional clinical understanding regarding HNC^21^. Saliva contains a range of different analytes including small organic metabolites and thereby, represents a resourceful biofluid for systemic information^22,23^. Moreover, simplicity of non-invasive sampling can facilitate repeated measurements without causing additional discomfort to the patient^24^. Saliva being proximal fluid in HNC, reflects both physiological and pathophysiological state of the disease. Thus, saliva as a biometrics is often utilised in several omics approaches including genomics^25,26^, transcriptomics^27,28^, proteomics^29,30^ and metabolomics^31,32^ for the discovery of HNC related biomarkers^33^. Eventually, there is a substantial gap of salivary volatilome use in clinical practices to assess the disease progression and monitor pathophysiology. Thus, analysing altered salivary volatilome in HNC patients is a practical approach to understand disease induced metabolic adaptations via identifying changes in concentrations of volatile organic metabolites (VOMs). Previously, we have evaluated the urinary volatile profiles from HNC cases using headspace solid phase microextraction (HS-SPME) approach coupled with gas-chromatography mass-spectrometry (GC-MS)^34^.

In the present study, we have demonstrated for the first time that HNC pathology driven changes in salivary volatilomic compositions can be efficiently extracted by HS-SPME and then quantified by GC-MS analysis. Here, we analysed volatile compositions of saliva in a cohort of 59 subjects (32 HNC patients and 27 controls) through our optimised HS-SPME GC/MS analytical setup. In this work, we aim to address the effects of HNC on saliva VOMs and if these effects were related to metabolic changes induced by HNC pathophysiology.

## Results

### Salivary volatilome identification by GC-MS

**Figure 1:**
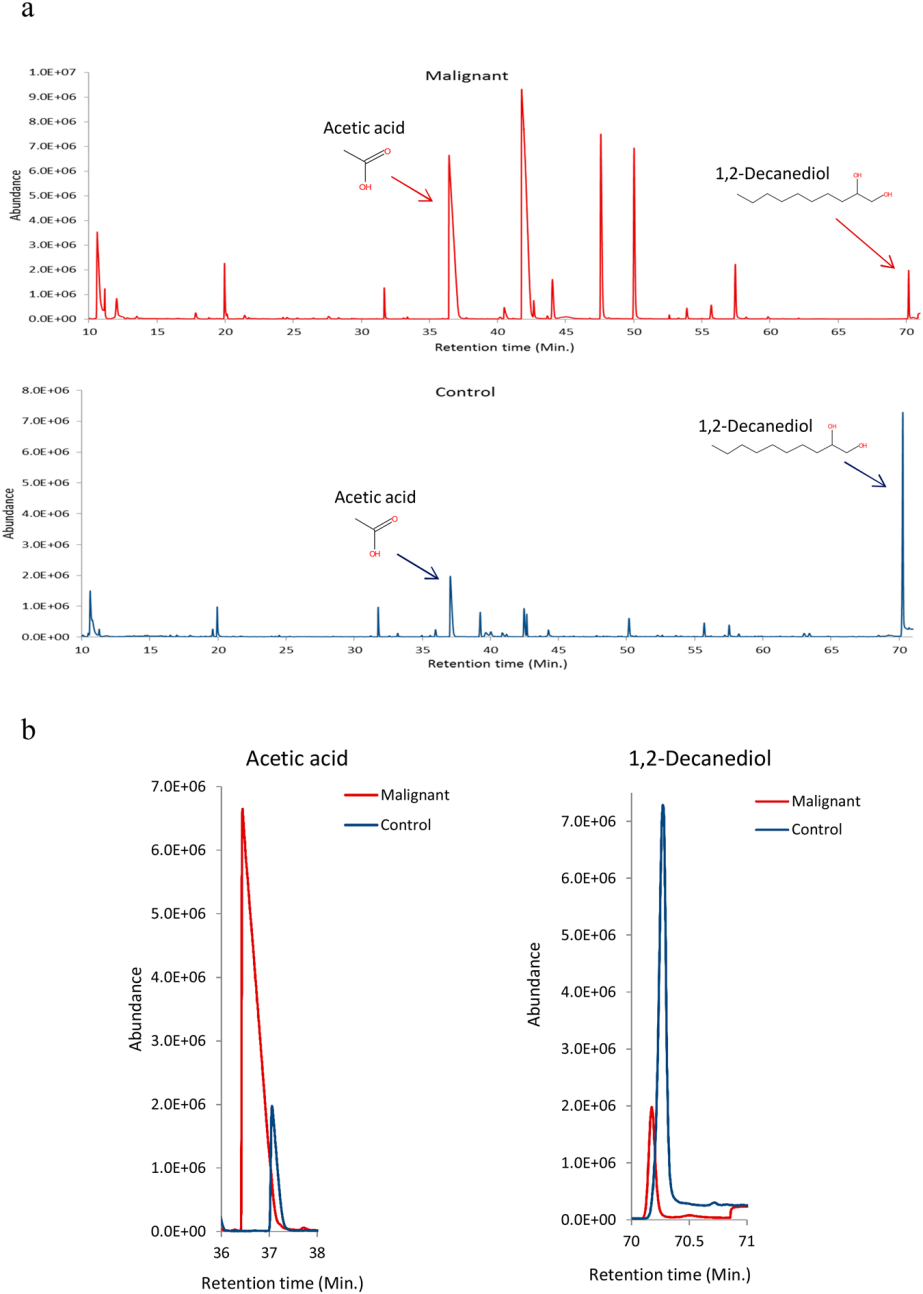
(**a**) Representative chromatogram of control and HNC subject, (**b**) Representative merged chromatogram depicting differential regulation of acetic acid and 1, 2-decanediol in HNC and control subject.

A total of 57 VOMs with ≥75% match with NIST library was identified in a cohort of 59 subjects, out of which, only 48 VOMs were finally considered for further statistical analysis (Supplementary Table S1). Those 9 VOMs were excluded as they were missing from >50% of samples. Representative chromatograms of HNC and control salivary VOMs are depicted in Fig. 1a. The identified VOMs belong to several chemical classes including aldehydes, ketones, alcohols, alkanes, organic acids, benzene derivatives and phenolic compound etc. As expected, some of the VOMs were differentially expressed in HNC as compared to controls, as shown in Fig. 1b. Furthermore, we have submitted our study to the MetaboLights database which can be accessed by ID MTBLS760.

### Identification of HNC specific salivary volatilome signature by multivariate statistical analysis

**Figure 2:**
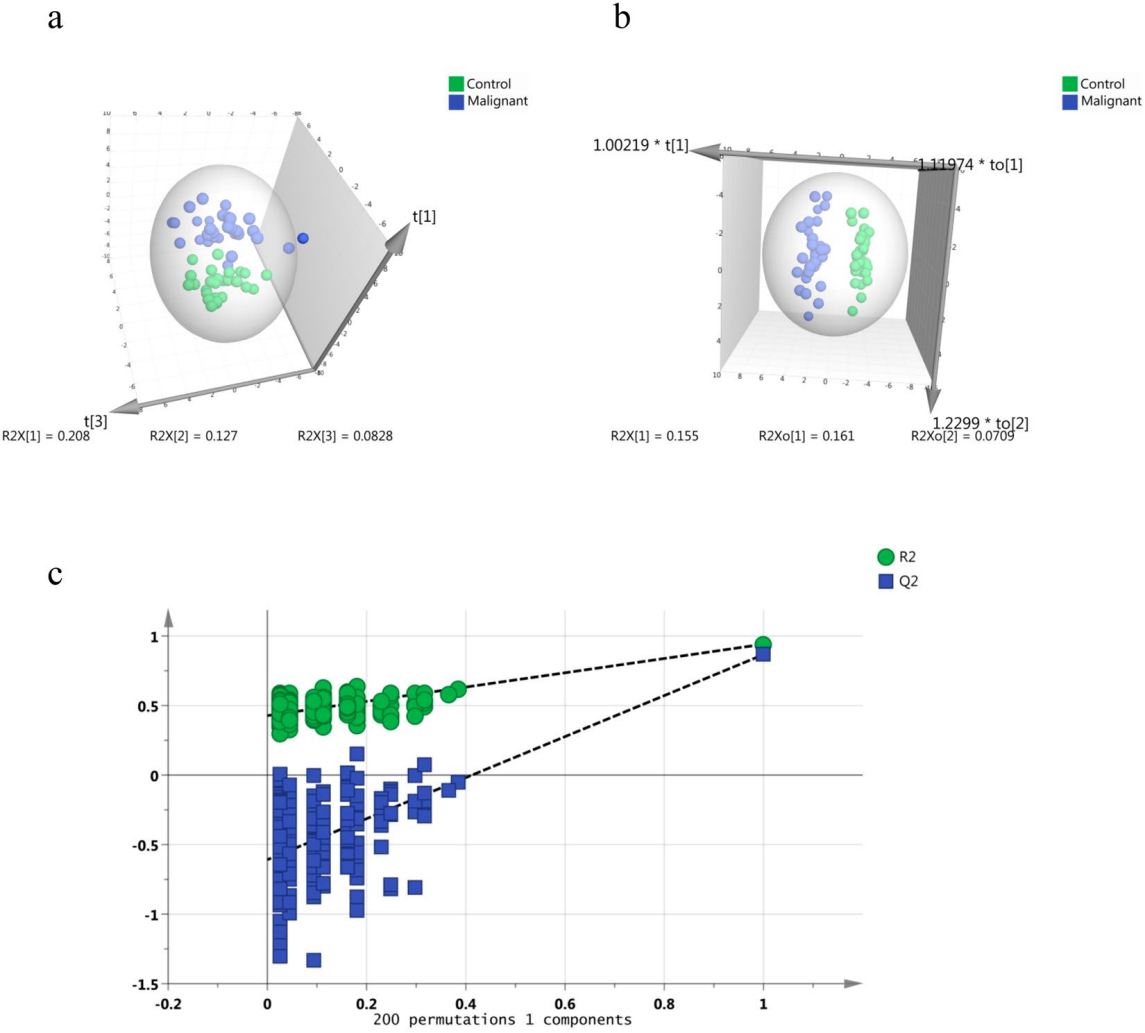
Analysis of altered salivary volatomic signatures by multivariate statistical tools. (**a**) Principal component analysis (PCA) of control and HNC subjects, (**b**) Orthogonal projections to latent structures discriminant analysis (OPLS-DA) of control and HNC subjects, (**c**) Permutation validation of OPLS-DA model. A total of 200 permutations were carried out to validate the model.

Overall, 27 VOMs of interest were selected via univariate statistical analysis such as wilcoxon rank sum test (*p* ≤ 0.05) along with their respective log_2_ FC values (≥0.58/≤−0.57), which are summarised in supplementary Table S2. Prominent group separation was observed in the principal component analysis (PCA) score plot revealing inherent concentration differences in salivary VOMs affected by HNC (Fig. 2a). Further enhanced group separation was achieved in orthogonal projections to latent structures discriminant analysis (OPLS-DA) model (Fig. 2b). Since the number of independent variables are higher than the sample size, statistical models such as OPLS-DA tends to over fit the data and give biased output or Voodoo correlations. Therefore, permutation validation (200 permutations) of OPLS-DA models was carried out to support its feasibility for our pilot approach (Fig. 2c). The permutation validation of OPLS-DA model yielded R2 and Q2 values of 0.941 and 0.867, respectively, indicating that the original model has high goodness of fit and good class prediction ability compared to permutated models and thus can be considered as a preliminary and realistic pilot model. VIP (variables important in projection) is regarded as criteria for selecting and ranking VOMs as per their contribution to class segregation in OPLS-DA score plot (Supplementary Fig. S1). Accordingly, a total of 15 VOMs were selected based on VIP > 1, FDR corrected *p*-value < 0.05, log_2_ FC value of ≥0.58/≤−0.58 and considered as most important differentially expressed metabolites responsible for class separation (Table 1). Some of the VOMs were validated by matching their fragmentation pattern as well as Kovats index with respective pure standard (Supplementary Table S3).

**Table 1:** The important discriminatory salivary VOMs selected by univariate and multivariate statistical analysis.

Sr. No.	Compound Name	HMDB ID	VIP score	*p*-value	FDR	Log_2_ FC
1	1,4-Dichlorobenzene	HMDB0041971	1.95	5.77E-11	2.77E-09	−4.46
2	1,2-Decanediol	—	1.76	7.48E-09	1.79E-07	−2.60
3	2,5-Bis1,1-dimethylethylphenol	—	1.59	8.32E-08	1.33E-06	−4.77
4	Propanoic acid, ethyl ester	HMDB0030058	1.45	1.06E-06	1.02E-05	3.38
5	E-3-Decen-2-ol	—	1.42	4.28E-07	5.13E-06	−2.34
6	Acetic acid	HMDB0000042	1.28	3.55E-06	2.84E-05	3.67
7	Propanoic acid	HMDB0000237	1.26	1.71E-05	0.000117	−1.36
8	Ethyl Acetate	HMDB0031217	1.21	0.000109	0.000524	1.86
9	2,4-Dimethyl-1-heptene	—	1.17	4.78E-05	0.000277	2.10
10	1-Chloro-2-propanol	—	1.15	0.002764	0.006318	1.74
11	1-Chloro-2-butanol	—	1.11	0.005423	0.011318	1.08
12	2-Propenoic acid	HMDB0031647	1.03	0.000135	0.000587	−0.81
13	2,3,3-Trimethylpentane	—	1.015	0.000333	0.001229	3.17
14	Ethanol	—	1.013	0.000674	0.002245	−1.00
15	1,2,3,4-Tetrachlorobutane	HMDB0000108	1.012	0.001216	0.003242	−0.81

Abbreviations: FDR- false discovery rate, VIP- variable importance in projection.

**Figure 3:**
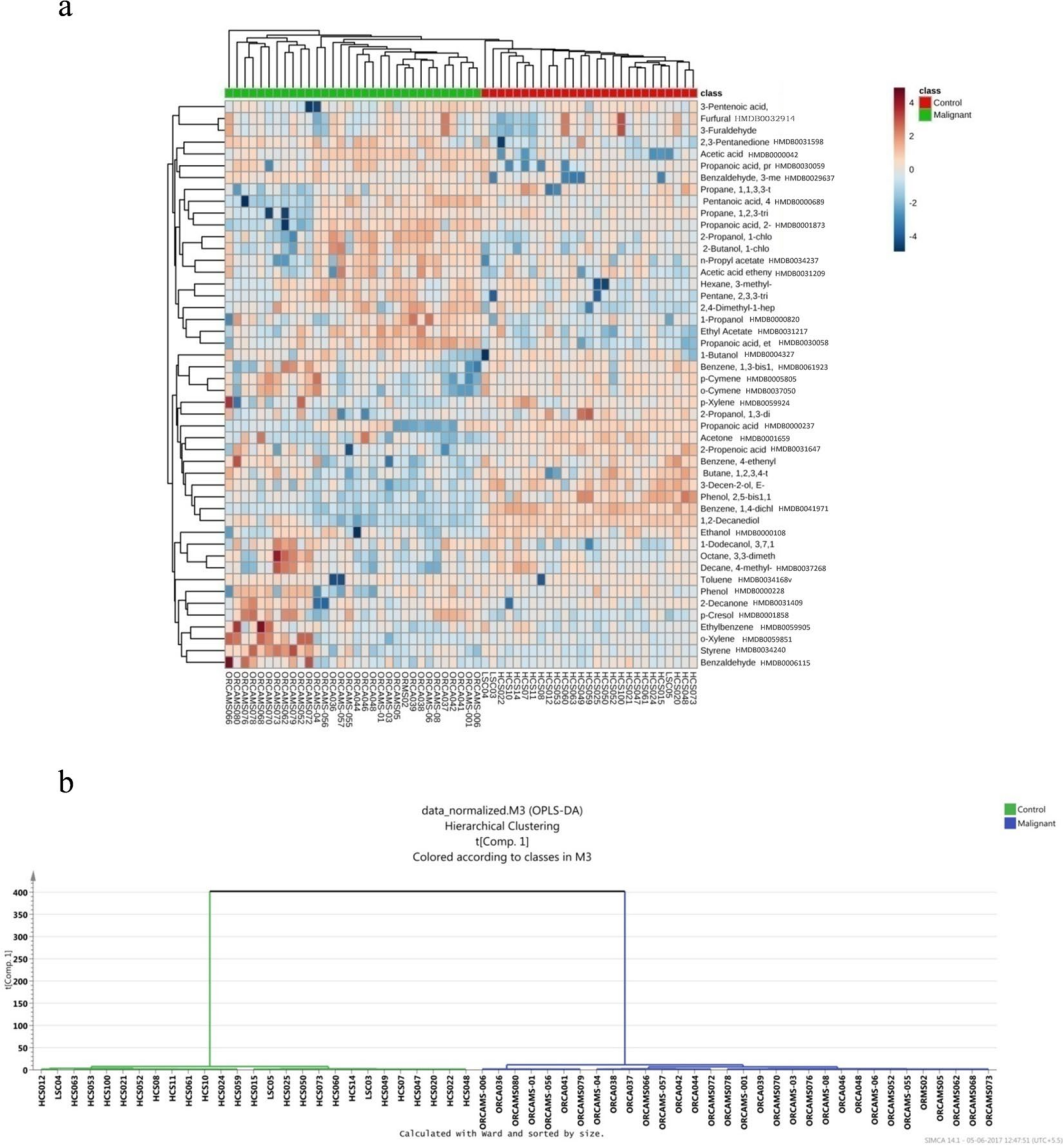
(**a**) Hierarchical clustering analysis of salivary volatilome shown as heat map and (**b**) dendrogram analysis of volatilomic data of HNC study subjects. The distance was calculated by ward’s method and sorted by size.

The heat map in Fig. 3a represents participant cohort related Hierarchical Cluster Analysis (HCA) clustering patterns. Each column in heat map indicates the metabolic pattern of individual subject in HNC and control cohort. Those colour patterns semi-quantitatively indicate varying alterations in salivary VOMs. A distinct clustering pattern associated with each study group was visible, indicating HNC specific intrinsic concentration differences in the VOMs. A dendrogram analysis had further segregated study subjects into their respective class and complemented initial HCA findings (Fig. 3b).

### Receiver operator characteristic (ROC) curve analysis of salivary VOMs

**Figure 4:**
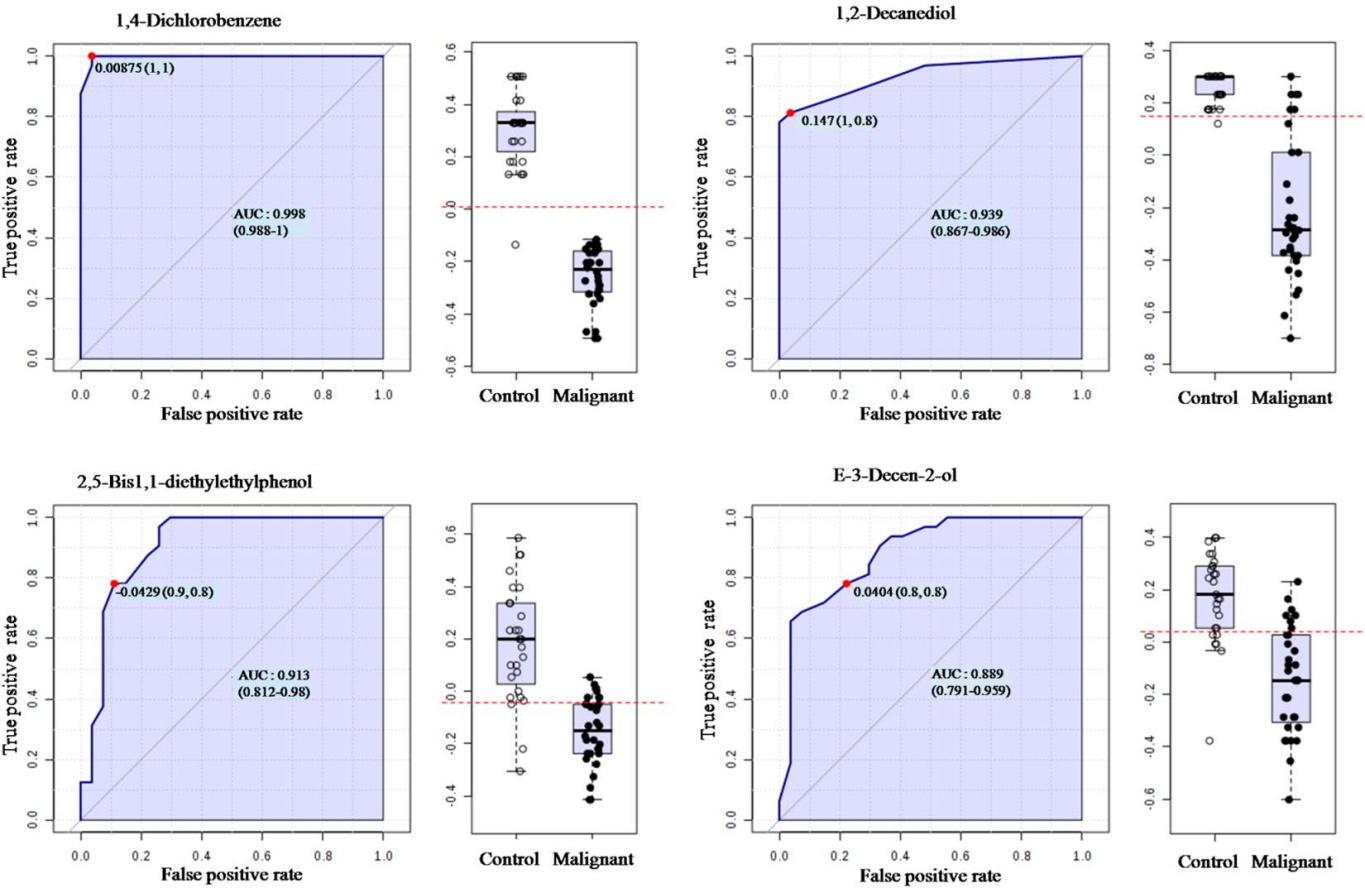
Receiver operator characteristic curve analysis of (**a**) 1,4-dichlorobenzene; (**b**) 1,2-decanediol; (**c**) 2,5-bis1,1-dimethylethyl and (**d**) E-3-decen-2-ol.

Among all the metabolites, we observed four VOMs with highest specificity and sensitivity for class segregation between study cohorts. These are 1,4-dichlorobenzene (AUC 0.998), 1,2-decanediol (AUC 0.939), 2,5-bis1,1-dimethylethylphenol (AUC 0.913) and E-3-decen-2-ol (AUC 0.889). Specificity, sensitivity and respective log_2_ FC with FDR corrected *p*-values of identified VOMs are depicted in Fig. 4 and Table 2.

**Table 2:** The salivary VOMs with highest specificity and sensitivity to discriminate HNC from control subjects.

Compound	HMDB	AUC	Sensitivity	Specificity	*p*-value	FDR	Log_2_ FC
1,4-Dichlorobenzene	HMDB0041971	0.998	1	1	5.77E-11	2.77E-09	−4.46
1,2-Decanediol	—	0.939	1	0.8	7.48E-09	1.79E-07	−2.60
2,5-Bis1,1-dimethylethyl- phenol	—	0.913	0.9	0.8	8.32E-08	1.33E-06	−4.77
E-3-Decen-2-ol	—	0.889	0.8	0.8	4.28E-07	5.13E-06	−2.34

Abbreviations: AUC- area under curve, FDR- false discovery rate.

### Identification of HNC altered metabolic pathways

**Figure 5:**
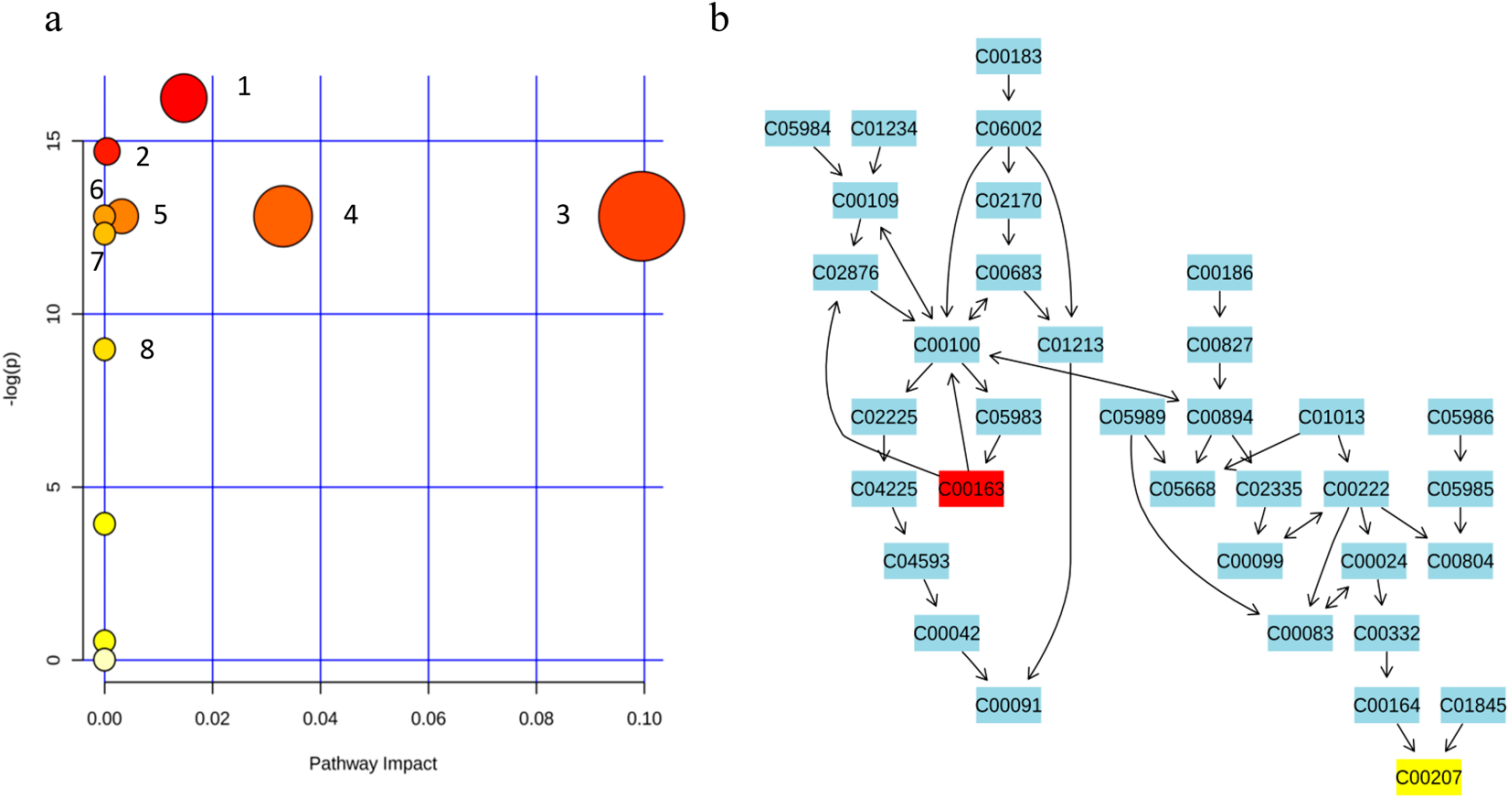
(**a**) Metabolic pathway analysis of salivary VOMs of HNC and control subjects. 1 – propanoate metabolism, 2 - glycolysis or gluconeogenesis, 3 - Pyruvate metabolism, 4–sulphur metabolism, 5 – selenoamino acid metabolism, 6 - taurine and hypotaurine metabolism, 7 - Nicotinate and nicotinamide metabolism, 8 - synthesis and degradation of ketone bodies, (**b**) Propanoate metabolic pathway. C00163 represents propanoic acid and C00207 represents acetone.

As indicated in Fig. 5, the metabolic pathways which are excessively active in HNC are glycolysis or gluconeogenesis, pyruvate metabolism, sulphur metabolism, selenoamino acid metabolism, taurine and hypotaurine metabolism, glycerolipid metabolism and tyrosine metabolism. We observed that nicotinate and nicotinamide metabolism was down regulated (further details are available in the Supplementary Table S4).

## Discussion

We observed profound effects of HNC on the volatile metabolite composition of human saliva. The VOMs identified in the saliva of study cohort belong to various chemical classes including organic acids, alcohols, phenolics, benzene derivatives and hydrocarbon etc. and those effects were also substance specific. Observed changes in VOM concentrations were significantly different in the biologically comparable but healthy control group. HNC induced changes were well attributed to *in vivo* metabolic effects induced by HNC pathophysiology.

Interestingly, metabolically demanding environments such as hypoxia and nutrient depletion induces significant metabolic reprogramming in cancer cells including enhanced glycolysis, lipogenesis and reduced dependence on oxidative phosphorylation, which is necessary to maintain a high rate of proliferation and exhibit malignant characteristics, such as altered cellular bioenergetics and metastatic behaviour^35,36^. It is important to note that the acetic acid and ethyl acetate were up-regulated in HNC subjects. It is well known that acetate serves as an alternative nutrient for cancer cells to support energy- and biomass production under hypoxia^37,38^. Moreover, acetate induces fatty acid synthesis as an immediate metabolic precursor and also functions as an epigenetic metabolite to promote cancer cell survival^39,40^. In fact, the enzyme nucleocytosol-localised acetyl CoA synthetase (ACSS2) involved in fatty acid biosynthesis is implicated as crucial enzyme for growth and survival of breast and prostate cancer cells cultured in hypoxic and low nutrient environment. This makes it an a attractive target for therapeutic studies^41^.

Genetic aberrations in malignant cells promote elevated oxidative stress, including high reactive oxygen species (ROS) production, which contributes significantly to lipid peroxidation of poly unsaturated fatty acids (PUFAs) and generates compounds such as 2,3,3-trimethylpentane and 1,2,3,4-tetrachlorobutane^42,43^. One of these alkanes, 2,3,3-trimethylpentane was found upregulated in the HNC patients compared to controls, which indicates a possible increase in ROS-mediated lipid peroxidation in HNC. In contrast, the other alkane, 1,2,3,4-tetrachlorobutane was found significantly reduced in HNC patients, which was most probably due to the conversion of this alkane to alcohol 1-chloro-2-butanol. Consequently, these alcohols were found in elevated concentrations. Such conversion can be mediated by enzymes like cytochrome P450, which are often induced during malignant conditions and hydroxylate alkanes generated during lipid peroxidation of PUFA^44^. Similarly, another alcohol “1-chloro-2-propanol” was also found at elevated concentration in presence of HNC.

Propanoic acid, a short chain fatty (SCF) acid mainly produced by anaerobic gut microbiota, showed down-regulation, while its ester derivative, ethyl ester propanoic acid (ethyl propionate), is significantly up-regulated in HNC cases. Overall, this reflects the up-regulation of propanoate under HNC. Propanoic acid is associated with a significant immunoregulatory activity and enhanced tissue sensitivity to insulin which makes it beneficial in the context of obesity and diabetes type 2. However, the mechanisms by which propanoate exert its effect on immunity and physiology in malignant conditions is still largely unknown^45,46^.

The metabolic pathway analysis revealed several up-regulated pathways in HNC subjects, including glycolysis or gluconeogenesis and pyruvate metabolism, which are in agreement with the higher aerobic glycolysis (i.e. Warburg effect) observed in cancer cells^47^. Similarly, it is well-known that hypotaurine is involved in the protection against oxidative stress and ROS generation, therefore, it is not surprising that taurine and hypotaurine metabolism is upregulated in HNC cases^48^. Finally, the upregulation of the Glycerolipid metabolic pathway in the HNC patients is also in accordance with a recent study showing that several genes in the glycerolipid metabolism are upregulated during tumorigenesis and metastasis^49^.

Taken together, our results have projected an unconventional understanding of HNC pathophysiology driven changes in cellular biochemistry and metabolic homeostasis. Once translated through rational clinical reasoning, this knowledge can substantially contribute to the understanding of several crucial aspects associated with HNC. Moreover, our observations could be accounted for several known/already investigated systemic phenomena (e.g. biochemical pathways). Our findings are novel and significantly important to warrant further investigations of disease driven metabolic effects through non-invasive follow-up of saliva and similar matrixes in the future. As there are insufficient references for retrospective frame in omics, prospective measurements and follow-up are mandatory to perceive a reasonable and rational clinical interpretation of differentially expressed VOMs as disease specific biomarkers.

## Conclusions

Our findings have provided a volatilomic insight into the salivary metabolite alterations under HNC. Further, clues from metabolic analysis indicated that short chain fatty acids might have significant role in HNC metabolism and bioenergetics. Though, the role of acetate in cancer cell metabolism is well scrutinised, the function of propanoate remains to be investigated further. These results therefore have contributed new basic and clinical knowledge in the field of HNC metabolomics and should foster multi-location clinical studies with larger cohorts to validate such findings. As VOMs inherits potential to be integrated in sensor-based Point of Care (PoC) applications, HNC prognosis and follow-up especially areas with limited modern healthcare access could be benefited in future.

## Materials and Methods

All experiments were performed in accordance to the Declaration of Helsinki (DoH) guidelines. The study was approved by the ethics committee of Armed Forces Medical College (AFMC) Pune and National Centre for Cell Science (NCCS), Pune. All the subjects voluntarily participated in the study after being informed about the investigation undertaken and written informed consent was obtained from them.

### Subject selection

Malignant Disease Treatment Centre (MDTC) of Military Hospital-Cardio Thoracic Centre (MH-CTC), Armed Forces Medical College (AFMC), Pune, India contributed towards collection of HNC saliva samples (n = 32). Control saliva samples (n = 27) were obtained at health check-up camps organised by the same hospital. Subject inclusion criteria for HNC were minimum 18 years old patient with histopathological confirmation of malignant lesion without any anticancer therapeutic intervention. Similarly, age and gender matched control subjects without hypertension, diabetes or any medication during last 3 months were recruited for this study. The clinical attributes of the study cohort are summarized in Supplementary Table S5.

### Sample collection

Saliva sampling was performed as recently reported^50^. Briefly, the saliva samples were collected during hospitalisation between 9.00 am to 12.00 at noon. Eating and drinking was not allowed at least 2 hours prior to sample collection. Each subject thoroughly rinsed their mouth with water and approximately 2 mL un-stimulated saliva was collected in 10 mL sterilised glass vial with screw cap and immediately placed on ice. All the samples were transported to the laboratory within 1 hour of sample collection and stored at −80 °C until further processing.

### Sample processing

Headspace - Solid Phase Microextraction (HS-SPME) approach was used for the extraction of the salivary VOMs. Detailed methodology for VOCs extraction from saliva was followed as per recently reported by Cavaco *et al*.^50^. Accordingly, samples were ice thawed and 1 mL saliva was transferred to a 4 mL headspace glass vial (Thermo Fisher, USA), acidified with 125 µL of 5 M HCL (Merck, Germany) and added 100 mg of NaCl (Merck, Germany). A magnetic stirring bar (0.5 × 0.1 mm) was also added to the vial that was closed with a Teflon (PTFE) septum screw cap. To concentrate the VOMs, a carboxen/polydimethylsiloxane (CAR/PDMS, 75 μm, Supelco, USA) SPME fibre was exposed to the headspace of the vial through the PTFE septa and incubated at 38 ± 1 °C for 45 min with continuous stirring at 800 rpm. After incubation, CAR/PDMS fibre was desorbed in the back-inlet port of the GC (250 °C) for 6 min. The CAR/PDMS fibre was preconditioned at 260 °C for 20 min to eliminate any residual VOMs from previous samples.

### GC-MS parameters and data pre-processing

Headspace extracted VOMs were analysed on BP-20 (SGE, Germany) fused silica capillary column (60 m × 0.25 mm × 0.25 µm) fitted to Agilent 7890B gas chromatograph (Palo Alto, USA) equipped with Agilent 5977 A quadrupole inert mass selective detector. The oven temperature programme was constituted as follows: initial temperature 45 °C held for 5 min, then increased to 150 °C with increment of 2 °C min^−1^ and followed by 10 min hold, then temperature was ramped up to 220 °C at the rate of 15 °C min^−1^ and further held for 15 min, with total run time of 87 min. Helium (99.999% purity, Prama Enterprises, India) was used as carrier gas at 1 mL min^−1^. The injections in splitless mode were performed with the back-inlet port maintained at 250 °C. The temperatures of transfer line, ionization source and quadrupole were maintained at 250 °C, 230 °C and 150 °C, respectively. After 5 min of solvent delay, data acquisitions were carried out in full scan mode in the range of 30 to 300 m/z and mass spectra were recorded at 70 eV. All the samples were acquired in duplicate and metabolites were identified by manual scrutiny of chromatograms by Chemstation software (Palo Alto, USA) equipped with NIST 11 library and by comparing mass spectra of pure standards when available. We have also carried out quality control samples (after every 10 samples) and blanks (after every 3 samples) to check the run to run variation as well as to detect any residual overhang of the previous samples. Metabolites were selected for further analysis based on match score of ≥75%. Metabolites missing from >50% of samples were removed from the final analysis.

### Statistical Analysis

In order to understand the influence of HCN on salivary volatiles, GC-MS derived peak area of each VOMs of interest were computed and treated by univariate and multivariate statistical methods. Features with <50% missing values were imputed by half of the minimum positive value in the data. The data was quantile normalised, transformed by log transformation and scaled by range scaling (Supplementary Fig. S2). Normalisation was carried out to remove the bulk differences among the samples in order to reduce the complexity of data and to make distribution of variables uniform across the samples so that the features can be more comparable.

At first, univariate statistical analysis i.e. wilcoxon rank sum test (a non-parametric *t*-test) was carried out to investigate differences in the concentrations of salivary VOMs in the subjects recruited. A FDR corrected *p*-value < 0.05 was used as criteria to select metabolites which are significantly altered in HNC subjects. Moreover, log_2_ FC of the identified VOMs was also carried out by calculating log_2_ transformation ratio of the mean metabolite abundance in HNC subjects relative to the control subjects. A log_2_ FC of≥ 0.58 is considered as a significant upregulation of the metabolite in HNC subjects while values ≤ −0.58 represents down regulation of metabolites. Data normalization and univariate statistical analysis as well as HCA, ROC curve and metabolic pathway analyses were carried out by Metaboanalyst 3.0^51^. Furthermore, to realize HNC affected salivary volatiles, multivariate statistical tools such as PCA and OPLS-DA were employed by using SIMCA 14.0 (Umetrics, Umea Sweden). At first, the PCA (an unsupervised multivariate statistical approach) was used to generate overview of data distribution across samples and detect possible outliers. Afterwards, a supervised multivariate statistical tool i.e. OPLS-DA was applied to enhance group separation. Moreover, the PCA was also plotted using the QC samples to ensure the data quality was not compromised (Supplementary Fig. S3).

HCA was performed using the concentration profile of each VOM to see clustering patterns associated with the participants. A heat map was generated using Minkowski distance measure and average algorithm. For dendrogram analysis, distance was measured by Ward algorithm and sorted by size. ROC curve analysis of salivary VOMs was performed to identify the varying metabolic effects on measured VOMs in diseased and control samples using MS peak areas.

### Metabolic pathways analysis

The salivary volatiolomic profiles of study subjects were used to get the overview of significantly dysregulated metabolic pathways in HNC with the help of MetPA tool of Metaboanalyst 3.0 web application.

## Electronic supplementary material

Supplementary Information
