# Supplementary material for: Volatilomic insight of head and neck cancer via the effects observed on saliva metabolites
Source: Sci Rep. 2018 Dec 7;8:17725. doi: 10.1038/s41598-018-35854-x (PMC6286361; doi:10.1038/s41598-018-35854-x)
Supplement: Supplementary file 1 — Supplementary Information [file 41598_2018_35854_MOESM1_ESM.pdf]

Volatilomic insight of head and neck cancer via the effects observed on saliva metabolites

Ravindra Taware[1], Khushman Taunk[1], Jorge A. M. Pereira[2], Amey Shirolkar[1], Dharmesh Soneji[3], José S. Câmara[2, 4], H A Nagarajaram[5, 6], Srikanth Rapole[1*]

1 *Proteomics Lab, National Centre for Cell Science (NCCS), Ganeshkhind, SPPU Campus, Pune 411007, India.*

2 *CQM – Centro de Química da Madeira, Universidade da Madeira, Campus Universitário da Penteada, Funchal 9000-390, Portugal.*

3 *Malignant Disease Treatment Centre, Military Hospital (Cardio Thoracic Centre), Armed Forces Medical College, Pune 411040, India.*

4 *Faculdade de Ciências Exatas e da Engenharia, Universidade da Madeira, Campus Universitário da Penteada, Funchal 9000-390, Portugal.*

5 *Laboratory of Computational Biology, Centre for DNA fingerprinting & Diagnostics (CDFD), Nampally, Hyderabad, 500001, India.*

6 *Department of Biotechnology & Bioinformatics, School of Life Sciences, University of Hyderabad, Hyderabad 500046, India.*

[*]Correspondence:rsrikanth@nccs.res.in

Table S1: Qualified VOMs for the statistical analysis

Sr.No.	VOMs	HMDB	Average ± RSD (%)	
			Control	HNC
1	Hexane, 3-methyl-	-	209848.23±96.47	853820.59±91.88
2	Pentane, 2,3,3-trimethyl-	-	191113.22±63.97	1671970.07±99.61
3	Acetone	HMDB0001659	3351820.48±39.41	15638723.05±238.16
4	2,4-Dimethyl-1-heptene	-	237582.7±111.18	1488804.54±143.86
5	Ethyl Acetate	HMDB0031217	1064881.76±129.01	14079238.72±117.34
6	Ethanol	HMDB0000108	36309995.09±146.04	38804522.44±114.03
7	Propanoic acid, ethyl ester	HMDB0030058	404522.88±118.31	7509760.89±109.74
8	n-Propyl acetate	HMDB0034237	945487.33±78.88	8575501.09±131.65
9	Acetic acid ethenyl ester	HMDB0031209	1222952.64±84.94	10507797.18±140.99
10	Octane, 3,3-dimethyl-	-	211910.53±81.48	983328.71±183.41
11	Decane, 4-methyl-	HMDB0037268	456727.94±81.82	1160677.32±152.03
12	Toluene	HMDB0034168	343831.92±42.5	753127.68±83.26
13	1-Propanol	HMDB0000820	466599.95±76.43	4373368.47±230.32
14	Propanoic acid, propyl ester	HMDB0030059	106081.97±232.7	390329.45±119.44
15	2,3-Pentanedione	HMDB0031598	263913.68±223.78	924131.12±169.22
16	1-Dodecanol, 3,7,11-trimethyl-	-	73733.76±66.78	154410.55±100.16
17	Ethylbenzene	HMDB0059905	354487.92±85.16	1038314.92±146.54
18	o-Xylene	HMDB0059851	321011.72±51.78	3012362.93±196.3
19	p-Xylene	HMDB0059924	964804.89±59.42	2251679.5±223.64
20	3-Decen-2-ol, (E)-	-	268795.62±60.32	135551.41±94.8
21	1-Butanol	HMDB0004327	1298337.01±69.29	7181423.66±116.09
22	Styrene	HMDB0034240	250922.15±33.25	1504878.04±128.11
23	p-Cymene	HMDB0005805	2090312.9±197.4	5326832.7±276.12
24	o-Cymene	HMDB0037050	1944846.71±209.54	5732404.56±270.54
25	2-Propanol, 1-chloro-	-	147589.86±43.86	723037.44±108.7
26	2-Butanol, 1-chloro-	-	367962.15±53.88	1287853.26±89.51
27	Propane, 1,2,3-trichloro-2-methyl-	-	212430.68±79.7	365165.77±67.78
28	Benzene, 1,3-bis(1,1-dimethylethyl)-	HMDB0061923	604897.71±36.95	1179780.98±130.35
29	Benzene, 4-ethenyl-1,2-dimethyl-	-	446632.14±91.48	710440.6±203.97
30	Benzene, 1,4-dichloro-	HMDB0041971	3504114.66±62.53	772987.67±59.09
31	2-Decanone	HMDB0031409	73567.04±57.74	1284990.33±255.48
32	Acetic acid	HMDB0000042	2556000.42±199.26	143445221±94.21
33	Furfural	HMDB0032914	419308.54±119.08	793543.96±152.71
34	3-Furaldehyde	-	434106.25±119.76	829336.12±153.07
35	Benzaldehyde	HMDB0006115	568686.25±36.84	2588791.95±247.25
36	Propanoic acid	HMDB0000237	21980444.65±84.44	79486430.6±140.58
37	Propane, 1,1,3,3-tetrachloro-2-methyl-	-	619791.68±57.92	567746.58±65.34
38	Propanoic acid, 2-methyl-	HMDB0001873	1207095.5±65.95	64107237.3±116.29
39	3-Pentenoic acid, 4-methyl-	-	268798.91±62.04	544092.85±109.46
40	2-Propenoic acid	HMDB0031647	877833.37±27.7	1110269.42±72.8

41	Benzaldehyde, 3-methyl-	HMDB0029637	61377.29±113.32	155555.68±139.61
42	2-Propanol, 1,3-dichloro-	-	547578.47±52.91	850767.71±102.91
43	Pentanoic acid, 4-methyl-	HMDB0000689	1534761.63±147.25	83352927.25±164.68
44	Butane, 1,2,3,4-tetrachloro-	-	143350.8±32.93	102692.46±77.42
45	Phenol	HMDB0000228	10639988.87±80.78	93602196.9±122.65
46	1,2-Decanediol	-	41547889.13±51.01	5220566.88±130.81
47	p-Cresol	HMDB0001858	268855.84±128.9	5399673.03±194.56
48	Phenol, 2,5-bis(1,1-dimethylethyl)-	-	3279454.57±118.51	599498.44±92.22

Table S2: VOMs identified by Wilcoxon-rank sum test and their respective log_2 fold changes.

Sr. No.	Compound Name	HMDB	*p*-value	FDR	Log_2 FC	Chemical class
1	Benzene, 1,4-dichloro-	HMDB0041971	5.77E-11	2.77E-09	-4.46	Benzene derivative
2	1,2-Decanediol	-	7.48E-09	1.79E-07	-2.60	Alcohol
3	Phenol, 2,5-bis1,1-dimethylethyl-	-	8.32E-08	1.33E-06	-4.77	Phenol derivative
4	3-Decen-2-ol, E-	-	4.28E-07	5.13E-06	-2.34	Alcohol
5	Propanoic acid, ethyl ester	HMDB0030058	1.06E-06	1.02E-05	3.38	Organic acid
6	Acetic acid	HMDB0000042	3.55E-06	2.84E-05	3.67	Organic acid
7	Propanoic acid	HMDB0000237	1.71E-05	0.000117	-1.36	Organic acid
8	2,4-Dimethyl-1-heptene	-	4.78E-05	0.000277	2.10	Alkene
9	Ethyl Acetate	HMDB0031217	0.000109	0.000524	1.86	Acid ester
10	2-Propenoic acid	HMDB0031647	0.000135	0.000587	-0.81	Organic acid
11	Pentane, 2,3,3-trimethyl-	-	0.000333	0.001229	3.17	Alkane
12	Ethanol	HMDB0000108	0.000674	0.002245	-1.00	Alcohol
13	p-Cresol	HMDB0001858	0.000701	0.002245	4.75	Phenol derivative
14	p-Xylene	HMDB0059924	0.000759	0.002276	0.95	Hydrocarbon
15	Propanoic acid, propyl ester	HMDB0030059	0.000853	0.002409	1.97	Organic acid
16	Butane, 1,2,3,4-tetrachloro-	-	0.001216	0.003242	-0.81	Alkane
17	Hexane, 3-methyl-	-	0.002044	0.005164	1.97	Hydrocarbon
18	2-Propanol, 1-chloro-	-	0.002764	0.006318	1.74	Alcohol
19	2-Propanol, 1,3-dichloro-	-	0.003796	0.008283	-0.89	Alcohol
20	2-Butanol, 1-chloro-	-	0.005423	0.011318	1.08	Alcohol
21	Benzaldehyde, 3-methyl-	HMDB0029637	0.005746	0.011493	0.69	Aldehyde
22	Propane, 1,1,3,3-tetrachloro-2-methyl-	-	0.006406	0.0123	-1.09	Alkane
23	1-Propanol	HMDB0000820	0.007012	0.012945	1.89	Alcohol
24	Benzene, 1,3-bis (1,1-dimethylethyl)-	HMDB0061923	0.009018	0.016031	0.80	Benzene derivative
25	2-Decanone	HMDB0031409	0.011136	0.01909	5.01	Ketone
26	o-Cymene	HMDB0037050	0.021877	0.03621	0.83	Benzene derivative
27	1-Dodecanol, 3,7,11-trimethyl-	-	0.028416	0.045466	0.92	Alcohol

Supplementary Figure 1:

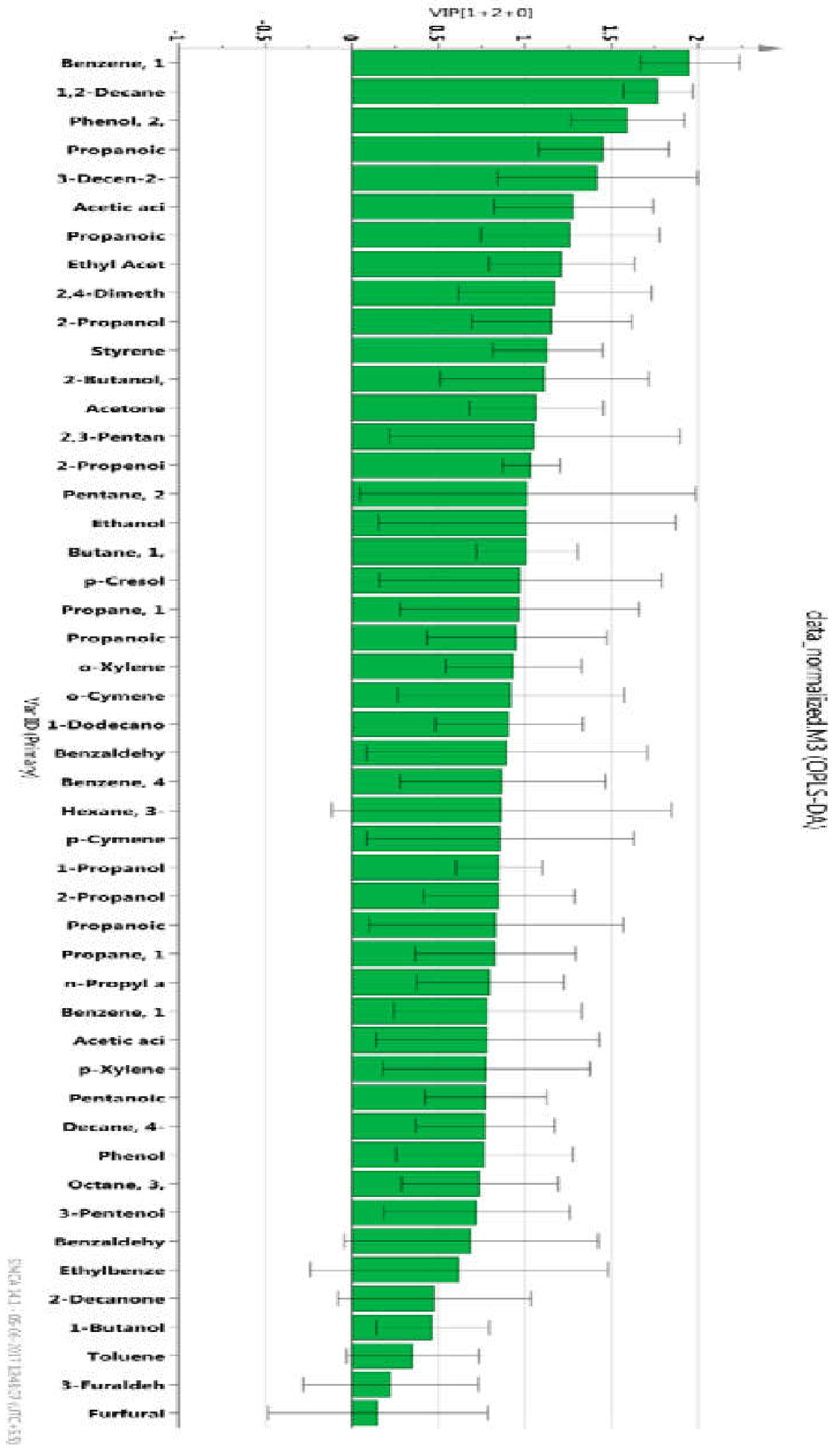

Figure S1: Variable Important in Projection (VIP) extracted from the Orthogonal Projections to Latent Structures - Discriminant Analysis score plot (OPLS-DA). VIP score of >1 used to select the VOMs contributing significantly to the class separation in the score plot of OPLS-DA.

Table S3: Validation of VOMs by matching fragmentation pattern and Kovats index.

Sr. No.	Compound	HMDB	Fragmentation (experimental)	Fragmentation (observed)	KI (exp)	KI (teo)
1	Benzene, 1,4-dichloro-	HMDB0041971	146/148/111/75	146/148/111/75	1372	1420
2	1,2-Decanediol		69/83/55/43	69/83/55/143	1945	-
3	Acetic acid	HMDB0000042	43/45/60/42	43/45/60/42	1396	1400
4	Propanoic acid	HMDB0000237	74/73/45/57	74/45/73/57	1472	1486
5	Ethyl Acetate	HMDB0031217	43/61/70/45	43/61/70/45	923	920
6	2-Propenoic acid	HMDB0031647	72/55/45/44	72/55/45/44	1568	-
7	Ethanol	HMDB0000108	45/46/43/42	31/45/46/43	948	947

Table S4: Pathway analysis of salivary volatile metabolites of HNC patients.

**Pathway**	**Total cmpd**	**Hits (log_2 FC)**	***p*-value**	**FDR**
Propanoate metabolism	35	Propionic acid (-1.36) ,Acetone (NS)	8.87E-08	9.76E-07
Glycolysis or Gluconeogenesis	31	Ethanol (-1.00), Acetic acid (3.67)	4.14E-07	2.27E-06
Pyruvate metabolism	32	Acetic acid (3.67)	2.70E-06	4.95E-06
Sulfur metabolism	18	Acetic acid (3.67)	2.70E-06	4.95E-06
Selenoamino acid metabolism	22	Acetic acid (3.67)	2.70E-06	4.95E-06
Taurine and hypotaurine metabolism	20	Acetic acid (3.67)	2.70E-06	4.95E-06
Nicotinate and nicotinamide metabolism	44	Propionic acid (-1.36)	4.44E-06	6.98E-06
Synthesis and degradation of ketone bodies	6	Acetone (NS)	0.000126	0.000173
Glycerolipid metabolism	32	Propyl alcohol (1.89)	0.019434	0.023753
Tyrosine metabolism	76	Phenol (0.69)	0.57944	0.63739
Butanoate metabolism	40	1-Butanol (NS)	0.98482	0.98482

Table S5: Demographic and clinical information of study cohort.

Particulars	HNC	Healthy control
Biological Specimen	Saliva	Saliva
Number of participants (male, female)	32 (19,13)	27 (15,12)
Age, median (range)	60 (36-82)	55 (33-75)
CaBuccalmucosa, (male, female)	9 (7, 2)	n.a.
Ca Tongue (male, female)	6 (2, 4)	n.a.
Ca Oral cavity (male, female)	9 (5, 4)	n.a.
Ca Alveolus (male, female)	3 (0, 3)	n.a.
Ca Maxilla (male, female)	2 (0, 2)	n.a.
CaGingivo labial sulcus (male, female)	2 (2, 0)	n.a.
Ca Central arch of mandible (male, female)	1 (1,0)	n.a.
Tobacco smoking (male, female)	8 (8, 0)	8 (8, 0)

Supplementary Figure 2:

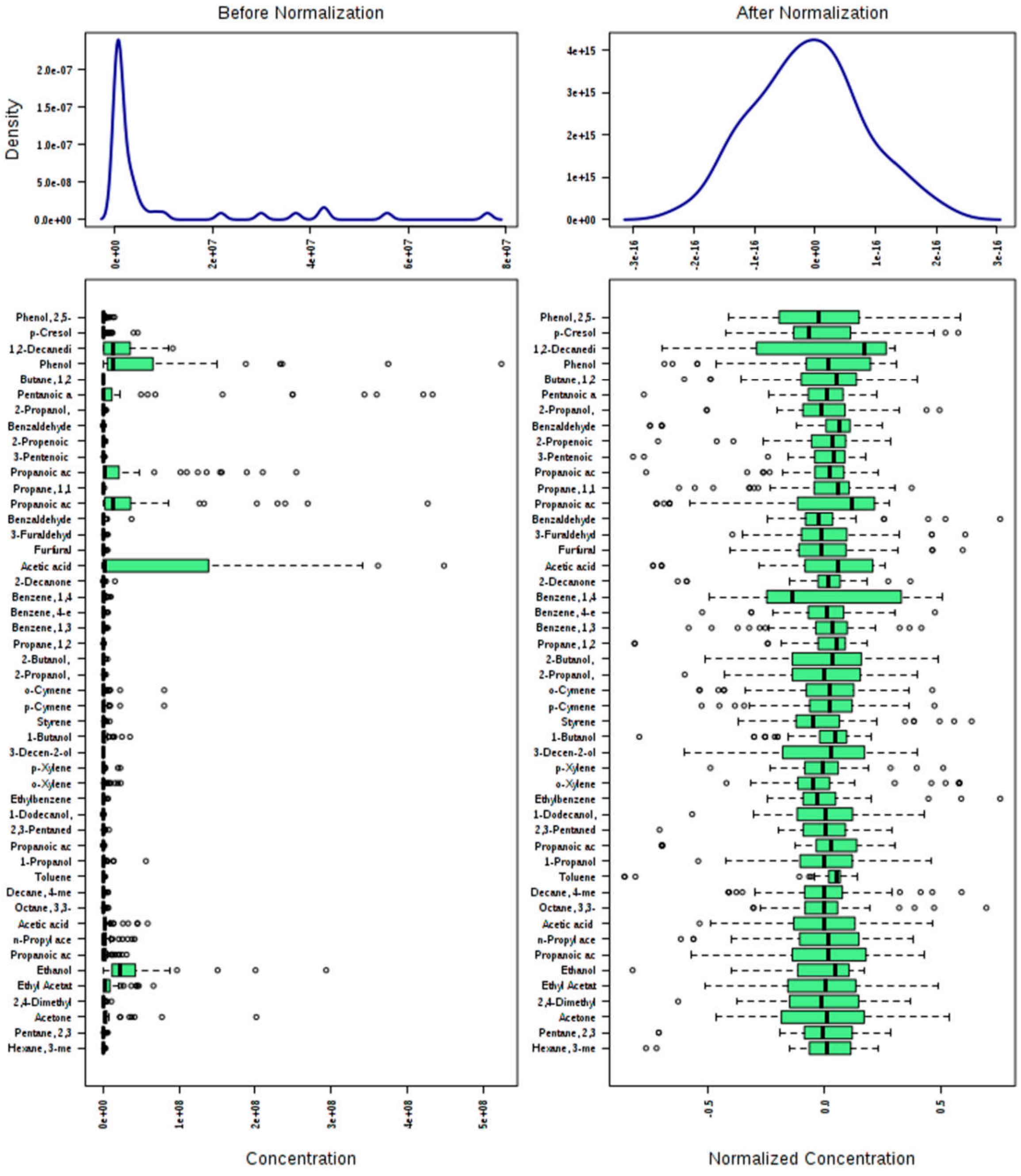

Figure S2: Representation of box plot and kernel density plot before and after data normalization. Selected methods: Row-wise normalization: Quantile normalization; Data transformation: Log transformation; Data scaling: Range scaling.

Supplementary Figure 3:

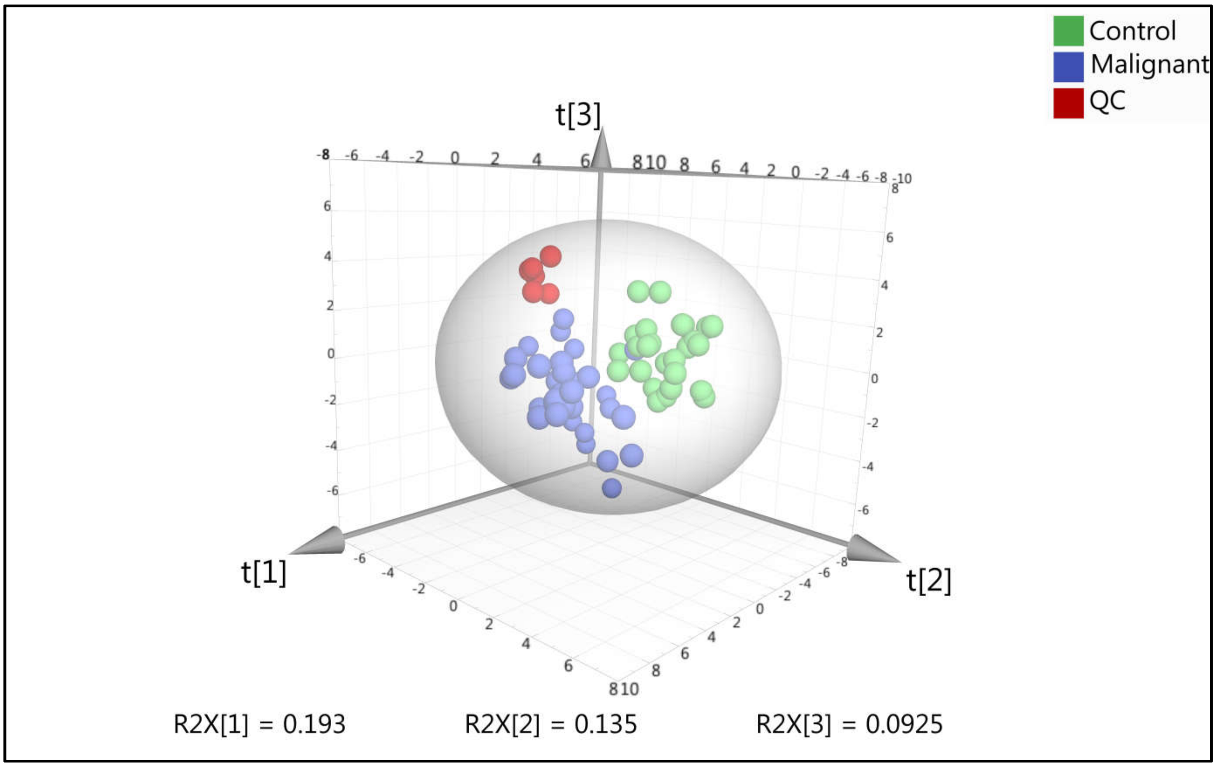

Figure S3: 3D graphical representation of PCA plot of QC samples along with control and HNC samples.
